# Identification of critical process parameters and quality attributes for bioreactor-based expansion of human MSCs

**DOI:** 10.3389/fbioe.2025.1608194

**Published:** 2025-08-21

**Authors:** Laura Herbst, Bastian Nießing, Robert H. Schmitt

**Affiliations:** ^1^ Department Bio-Adaptive production, Fraunhofer Institute for Production Technology (FHG), Aachen, Germany; ^2^ WZL RWTH Aachen, Chair of Intelligence in Quality Sensing, Laboratory for Machine Tools and Production Engineering, RWTH Aachen University, Aachen, Germany

**Keywords:** quality-by-design, critical process parameter, critical quality attributes, mesenchymal stem cells, standardization, bioreactor

## Abstract

Mesenchymal stem/stromal cells (MSCs) have been identified as a promising therapeutic option for osteoarthritis, graft vs. host disease and cardiovascular diseases, among others. For widespread application of these therapies, robust and scaled manufacturing processes are required that reliably yield high amounts of high quality MSCs. One of the primary challenges in MSC manufacturing is achieving robustness, due to the high donor-to-donor and batch-to-batch variability seen in MSC manufacturing. To achieve more consistent manufacturing, standardization of the manufacturing process and analytical methods to determine cell quality and control process parameters will be needed. Traditionally, MSCs are cultivated in two dimensional (2D) systems, such as flasks or plates. However, these systems are limited in their scalability. To enhance volumetric productivity, upscaling may be achieved using agitated bioreactors where the MSCs are grown on microcarriers or other types of scaffolds. In this article, we have reviewed existing publications on the manufacturing of MSCs in agitated bioreactor systems regarding the process conditions used and the quality parameters measured to define more clearly the most relevant cell quality and process parameters. Key cell quality parameters measured are cell number and viability, immunophenotype and differentiation potential, while key process parameters include the cultivation system (cell source, bioreactor type, media composition), physiochemical properties of the media such as pH and dissolved oxygen (DO), as well as nutrient supply. Defining these parameters more clearly will support the development of robust MSC manufacturing processes at scale using improved process control and facilitate the widespread clinical application of MSC-based cell therapies.

## Introduction

The lack of curative treatments for widespread diseases such as osteoarthritis, cardiovascular diseases, and autoimmune deficiencies has led to the rise of Advanced Therapy Medicinal Products (ATMPs). Among these, mesenchymal stem/stromal cells (MSCs) are considered a promising treatment for many therapeutic applications, such as osteoarthritis, graft *versus* host disease (GvHD), and cardiovascular diseases (García-Gómez et al., 2010; Rodríguez-Fuentes et al., 2021). Globally, there are twelve approved MSC cell therapies with nine originating from Asia. In Europe, two products were approved by the European Medicines Agency (EMA): Holoclar, which facilitates corneal epithelium repair (European Medicines Agency, 2021), and Alofisel, used for treating complex fistulas in Crohn’s disease (European Medicines Agency (EMA)), whose authorisation was withdrawn by request of the marketing-authorisation holder. In the United States, one therapy has been approved by the Food and Drug Administration (FDA): Remestemcel-L for treating GvHD (RYONCIL, 2025). As more MSC therapies advance through clinical development, the demand for MSC manufacturing is expected to rise significantly. MSCs are multipotent, primary cells, which can be extracted from different tissue sources, such as bone marrow, umbilical cord, or adipose tissue, or differentiated from induced pluripotent stem cells (iPSCs) (Wruck et al., 2021; Pittenger et al., 1999).

For widespread application of MSCs as a therapy, the manufacturing must become more industrialized and overcome several critical challenges (de Almeida Fuzeta et al., 2020; García-Fernández et al., 2020). One limitation is the prevalent use of two-dimensional cultivation systems for MSC expansion, which restricts scalability. Transitioning to three-dimensional bioreactor systems can enhance productivity and allow for greater scalability (Tsai and Pacak, 2021). Maintaining quality during this scale-up process is essential to ensure therapeutic efficacy. Secondly, as MSCs are extracted from varying tissue sources, they are subject to substantial biological variability stemming from the tissue source and the donor (Costa et al., 2021; Győrgy et al., 2019; Kanawa et al., 2013; Mareschi et al., 2006; Pachón-Peña et al., 2016; Sammour et al., 2016). This has led to an effort to better characterize the extraction and cultivation process of MSCs. However, further standardization is needed for the cultivation process and the process metrology involved as MSC heterogeneity remains a challenge for clinical translation (Costa et al., 2021; Nikolits et al., 2021). Thirdly, this variability in MSC growth and phenotype necessitates a well characterized and standardized method for quality control (Silva Couto et al., 2020). The International Society for Cell and Gene Therapy (ISCT) defined minimal criteria for MSCs in 2009 as being plastic adherent, must express CD105, CD73 and CD90, and lack expression of CD45, CD34, CD14 or CD11b, CD79α or CD19 and HLA-DR surface markers and must differentiate to osteoblasts, adipocytes and chondroblasts *in vitro* (Dominici et al., 2006). This definition was recently updated as part of an expert survey (Renesme et al., 2025).

The regulatory pathway for the approval of an MSC product differs by region, Europe, US, Canada, Asia and regulatory authority (López-Beas et al., 2020). To engineer a pharmaceutical processes with a primary focus on product quality, the International Council for Harmonization (ICH) introduced the Quality-by-Design approach in 2009 (EMA, 2009). The guideline Q8 details a scientific and risk-based approach, where the desired product quality is detailed early in the product development and the focus of process development is set on meeting this defined quality efficiently. In a first step, a Quality Target Product Profile (QTPP) is defined, which is used as a basis to specify critical quality attributes (CQA). CQAs are key product parameters which must be met to ensure the drug product’s safety and efficacy. Thirdly, existing process understanding is utilized, and the production process is systematically evaluated to establish process controls ensuring CQAs are met throughout. QbD has been applied to the manufacturing of MSCs to identify the QTPP and define critical process parameters (CPP) (Sharma et al., 2014; Lipsitz et al., 2016; Maillot et al., 2021; Guadix et al., 2019). These studies suggest a QTPP of dosage (cell number and viability), Potency (Identity, differentiation potential and *in vivo* effect), and product quality (genetic stability, purity). The processes considered for these studies include the cell extraction and the two-dimensional expansion of MSCs, and give a list of CPPs for these unit operations (Maillot et al., 2021). As widespread application of MSCs will likely necessitate the expansion of MSCs in bioreactor systems, this review focuses on evaluating the expansion of MSCs in bioreactors in terms of the quality attributes and process parameters considered in current process development.

## Systematic literature review and analysis

The literature search for this review was carried out according to the PRISMA methodology (PRISMA statement). The search string “(stirred OR agitated) AND (bioreactor OR expansion) AND mesenchymal AND (stem OR stromal) AND cell” was entered in the web of science search engine. The results were filtered for original research articles, reviews and meeting abstracts were excluded (I). Only articles using human MSCs were considered, animal based MSCs, co-cultivation with other cell types and tissue culture are not considered (II). Only articles conducting experimental work were considered; simulations and models using data published elsewhere were excluded to avoid redundancies (III). A list of the articles and exclusion reasoning is given in the Supplementary S1 along a PRISMA chart in S2. A total of 142 articles were evaluated, with 76 articles being included in the following analysis. The list of quality attributes (S3) and process parameters (S4), along with their frequency and weightings in terms of implementation of costs and criticality to quality is given in the supplementary. A detailed description of the Pareto Analysis performed is given as well (S5).

## Quality attributes measured in bioreactor-based expansion of MSCs

**FIGURE 1:**
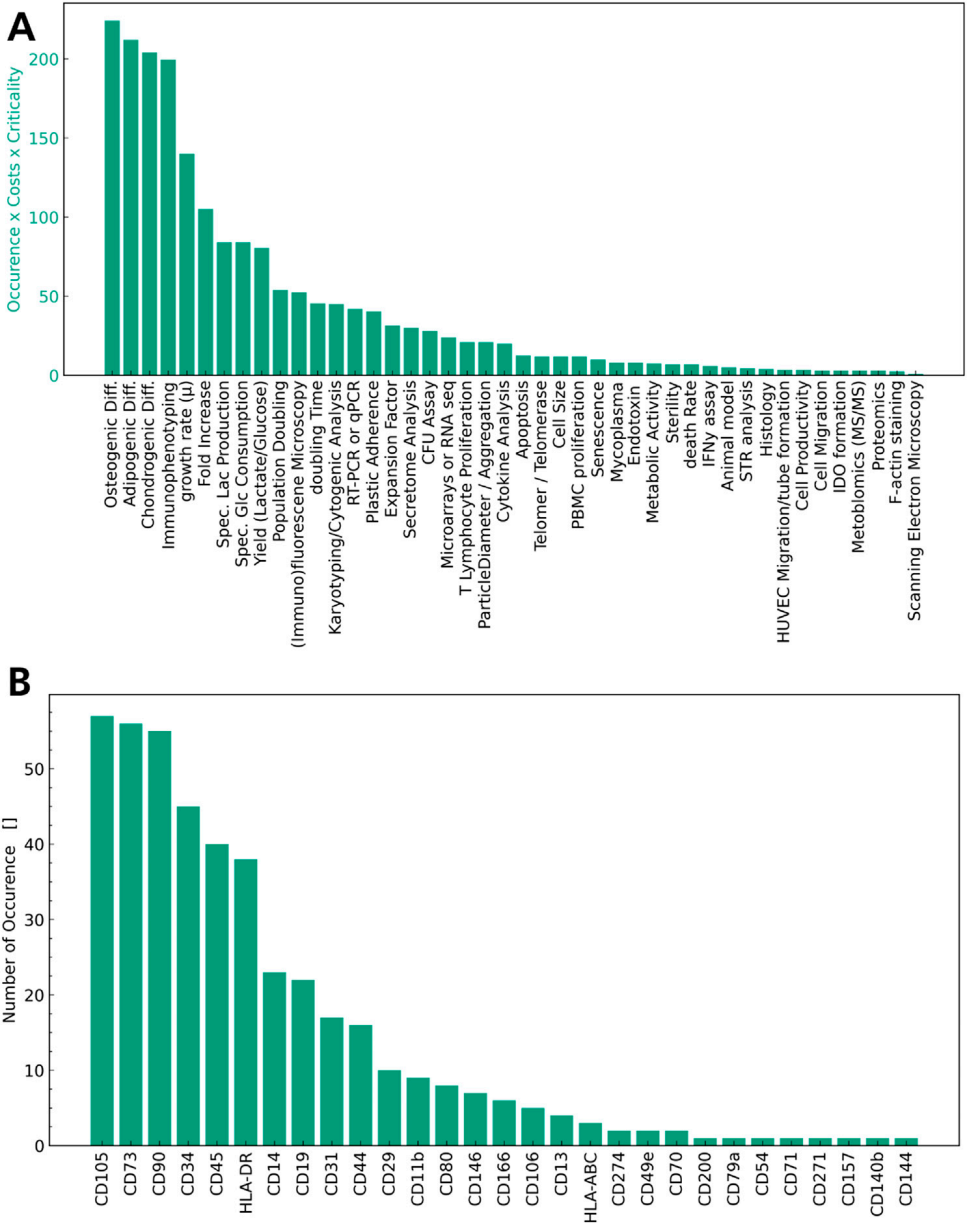
Quality attributes sorted by number of occurrences. In **(A)** All attributes characterising the immunophenotype are accumulated into one QA. **(B)** Surface markers measured as QA of MSCs by number of occurrences.

Of the 79 quality attributes recorded, 27 refer to the immunophenotype of the MSCs, while others characterise cell growth or health of the MSCs. The quality attributes sorted by number of occurrences and weighted according to S5 are given in Figure 1A.

Cell Count and viability are measured ubiquitously as quality attributes in MSC expansion in bioreactors in these articles (28–103) as illustrated in Figure 1A. Cell count and viability characterise the dosage of the final product, which is a well-established part of the QTPP for MSCs and across all cell-based therapies. It is also highlighted as a central aspect in the EMA Guideline on quality, non-clinical and clinical requirements for investigational ATMPs (European Medicines A gency (EMA)). Figure 1A illustrates the immunophenotype of the MSCs (Carmelo et al., 2015; Chen et al., 2015; Cunha et al., 2017; de Da Sá Silva et al., 2020; Dos Santos et al., 2014; Eibes et al., 2010; Gadelorge et al., 2018; Gao et al., 2023; Gonzalez Gil et al., 2020; Jorgenson et al., 2018; Kehoe et al., 2012; Kurogi et al., 2021; Kurogi et al., 2022; Leber et al., 2017; López-Fernández et al., 2024a; López-Fernández et al., 2024b; Mizukami et al., 2016; Moreira et al., 2020; Nienow et al., 2016; Noronha et al., 2021; Osiecki et al., 2015; Petry et al., 2016; Rafiq et al., 2016; Rafiq et al., 2013; Santos et al., 2011; Schirmai et al., 2014; Sion et al., 2021; Sousa et al., 2015; Sunil et al., 2013; Timmins et al., 2012; Tozetti et al., 2017; Yan et al., 2020; Wang et al., 2023; Yu et al., 2009; Yuan et al., 2014; Zhang et al., 2022; Zhang et al., 2023) as well as the differentiation capability into one or all three mesodermal lines (Carmelo et al., 2015; Chen et al., 2015; Cunha et al., 2017; de Da Sá Silva et al., 2020; Dos Santos et al., 2014; Dosta et al., 2020; Eibes et al., 2010; Gadelorge et al., 2018; Gao et al., 2023; Gonzalez Gil et al., 2020; Jorgenson et al., 2018; Lawson et al., 2017; Kurogi et al., 2021; Lam et al., 2017; Leber et al., 2017; López-Fernández et al., 2024a; López-Fernández et al., 2024b; Mizukami et al., 2016; Moreira et al., 2020; Nienow et al., 2016; Noronha et al., 2021; Osiecki et al., 2015; Petry et al., 2016; Rafiq et al., 2016; Rafiq et al., 2013; Rotondi et al., 2021; Santos et al., 2011; Sart et al., 2013; Sart et al., 2009; Sart et al., 2010; Schirmai et al., 2014; Sion et al., 2020; Sousa et al., 2015; Sunil et al., 2013; Timmins et al., 2012; Tozetti et al., 2017; Yan et al., 2020; Wang et al., 2023; Yu et al., 2009; Yuan et al., 2014; Zhang et al., 2022; Zhou et al., 2013) are measured frequently, concurring with the minimal criteria defined by the ISCT. Apart from phenotyping and differentiation potential, indices of growth, cell age and metabolic activity, such as the growth rate, fold increase, metabolic consumption and production rates, and yields are also used as seen in Figure 1A (Carmelo et al., 2015; Chen et al., 2015; Cunha et al., 2017; de Da Sá Silva et al., 2020; Dos Santos et al., 2014; Dosta et al., 2020; Eibes et al., 2010; Gao et al., 2023; Gonzalez Gil et al., 2020; Grein et al., 2016; Jorgenson et al., 2018; Lawson et al., 2017; Kehoe et al., 2012; Lam et al., 2017; Leber et al., 2017; López-Fernández et al., 2024a; López-Fernández et al., 2024b; Mizukami et al., 2016; Moreira et al., 2020; Nienow et al., 2016; Noronha et al., 2021; Osiecki et al., 2015; Petry et al., 2016; Rafiq et al., 2016; Rafiq et al., 2013; Santos et al., 2011; Sart et al., 2009; Sart et al., 2010; Schirmai et al., 2014; Sion et al., 2020; Sion et al., 2021; Sousa et al., 2015; Sunil et al., 2013; Tozetti et al., 2017; Yan et al., 2020; Wang et al., 2023; Yuan et al., 2014; Zhang et al., 2022; Zhou et al., 2013). A wide range of *in vitro* assays targeting a specific function of MSCs, such as the T Lymphocyte proliferation assay (Dosta et al., 2020; Elseberg et al., 2012; Gadelorge et al., 2018; Lawson et al., 2017; Kurogi et al., 2021; Mizukami et al., 2016; Noronha et al., 2021; Tozetti et al., 2017) to assess modulation of the immune system or apoptosis (Cunha et al., 2017; Gonzalez Gil et al., 2020; Schirmai et al., 2014; Sion et al., 2020; Sousa et al., 2015; Timmins et al., 2012) to assess cell health are considered, but not used frequently overall. Interestingly, though plastic adherence is a characteristic of MSCs according to the ISCT criteria, it is not commonly mentioned as a quality attribute for MSCs in the articles surveyed (Cunha et al., 2017; López-Fernández et al., 2024b; Nienow et al., 2016; Noronha et al., 2021; Rafiq et al., 2013). As the MSCs in these studies are typically grown on microcarriers, many of them exhibit plastic adherence without it being expressly stated as an attribute of their identity.

When considering identity and potency in the QTPP, the most prevalent methods to determine quality attributes are identified based on the literature analysis in Figure 1A. In terms of identity, immunophenotyping is used widely to characterise the MSCs and the absence of any undesirable cells. In Figure 1B the different surface markers measured across the articles reviewed are sorted by number of occurrence. When comparing these to the minimal criteria established by the ISCT, the positive markers (CD105, CD90, CD73) are determined in every study using immunophenotyping, while HLA-DR, CD45 and CD34 are the most prevalent negative markers used, with a number of less frequently used markers also shown in Figure 1B. These findings illustrate the degree of standardisation in measurement of positive markers with a lack of standardisation for negative surface markers. This limitation was addressed by the ISCT recently, suggesting to amend the definition to focus on the positive MSC markers (CD105, CD90, CD73) and allow for variation in the negative markers based on the cell source and application of MSCs, though CD45 was highlighted as the negative MSC marker to be used ubiquitously (Renesme et al., 2025). Overall immunophenotyping is a frequently used and suitable method to determine identity of the cells in an MSC product, and some degree of standardisation is observed.

A key attribute in terms of potency of MSCs is their multipotency and ability to differentiate into osteoblasts, chondrocytes, and adipocytes. This mechanism of action is intended to be used in treating osteoarthritis and other tissue defects and is determined by performing trilinear differentiation of MSCs *in vitro* post manufacturing. While this quality attribute characterises the differentiation capabilities, its application as a potency assay is limited. The differentiation capacity of MSCs is not the sole or main mode of action in many therapeutic MSC applications, whereas the immunomodulatory properties, the secretion of bioactive factors or any combination of the three may be utilised. To this extent the analysis of other attributes, such as characterisation of the transcriptome, immunomodulatory assays, DNA methylation profiling or secretome profiling have been suggested to additionally characterise MSCs (Kadri et al., 2023; Krampera et al., 2021; Wong et al., 2021). This variability is evident in the literature analysis performed as seen in Figure 1A, as a number of assays are listed such as the T lymphocyte or PBMC proliferation assay to assess immunomodulatory properties, secretome analysis to assess secretion of bioactive factors or Metabolomics, Proteomics or gene expression profiling to characterise cell function in depth. To this date, the discussion around increased standardisation of potency assays in the therapeutic applications of MSCs has not been conclusive (Renesme et al., 2025) concurring with the study conducted in Figure 1A. The potency of an MSC product in contrast to identity and safety is inherently difficult to standardise across therapeutic applications, as it ideally should align with the main mode of action of the MSCs. MSC therapeutics are explored across a wide range of indications, and this should be reflected in their respective potency assays. While the literature review conducted suggests differentiation capacity of MSCs as a CQA, differentiation capacity of MSCs alone is insufficient to elucidate potency of the MSC product and additional assays are needed.

With regard to safety, only a limited number of studies report on quality attributes relating to the genetic stability, such as karyotyping or the telomerase activity of the MSCs, with even less studies reporting on the sterility, endotoxin or *mycoplasma* analysis in the MSC product. Considering the regulatory frameworks, these parameters are crucial to the QTPP of MSC products. The absence of microbial contaminations must be determined for any ATMP. Similarly, the genetic stability and tumorgenicity are important risk factors in the application of MSCs and must be considered as CQAs (López-Beas et al., 2020; Maillot et al., 2021; European Medicines A gency (EMA)). For the EMA, assays to verify the absence of contaminants as well as genetic stability and tumorigenicity are expressly stated as part of the guidelines (European Medicines A gency (EMA)). This discrepancy in the literature review conducted is likely due to the articles reviewed focusing on earlier product and process development, where safety quality attributes are not ubiquitously measured yet.

## Process parameters measured in bioreactor based expansion of MSCs

**FIGURE 2:**
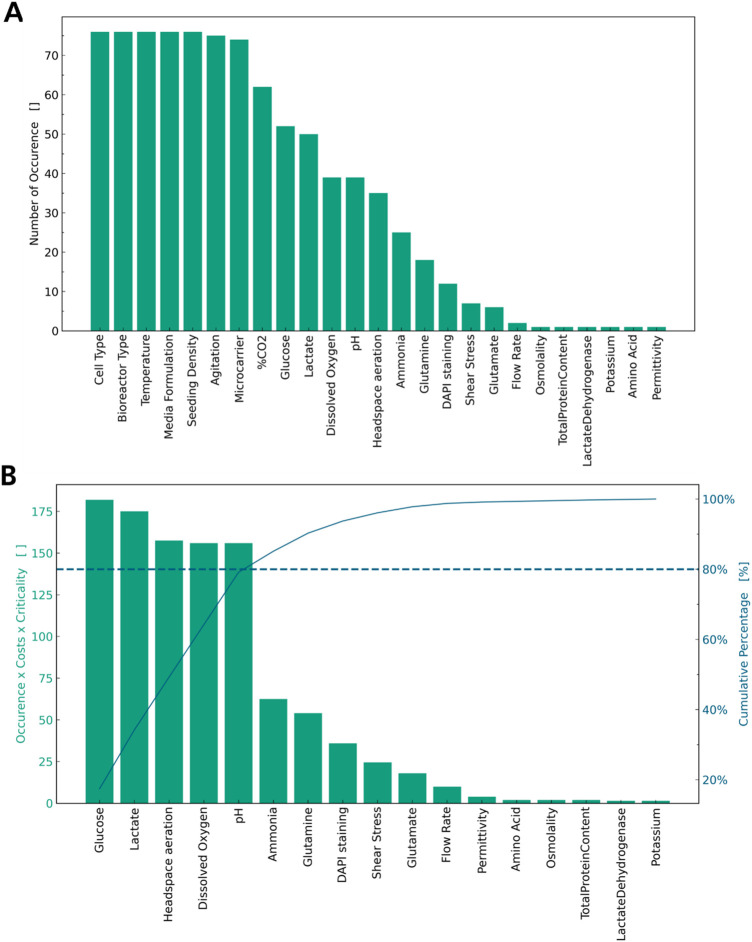
List of process parameters by number of occurrences. **(A)** Cell Type, Bioreactor Type, Media Formulation are accumulated. **(B)** Highly reoccurring parameters are dropped and a weighted Pareto Analysis is applied.

In addition to quality parameters, process parameters must be evaluated to successfully establish a process robustly yielding high-quality MSCs for cell therapy. In the literature analysis, 41 process parameters were identified overall. Ten of those refer to the origin of the MSCs used for the experiment, with the most frequently used cells being bone marrow derived MSCs, umbilical cord derived MSCs, and adipose derived MSCs (S3). Five of the process parameters listed refer to the type of bioreactor used for the experimental work, with spinner flasks and stirred tank bioreactors used most (S3). In Figure 2A, these parameters are summarised as “Cell Type” and “Bioreactor Type” respectively to facilitate visualisation, and the process parameters are sorted by the number of occurrences in the articles. The parameter “media formulation” refers not only to the type of basal media used, but also to the presence or absence of supplements, such as FBS or platelet lysate. As media formulation has been extensively reviewed elsewhere (Gottipamula et al., 2013; Bui et al., 2021; Doron and Temenoff, 2021), this is not a focus of this review. Similarly, “Microcarrier” summarises all types of microcarriers used in the different articles, as these have been evaluated previously (Leber et al., 2017; Rafiq et al., 2016). The parameter “amino acid” refers to any amino acid measured other than glutamine or glutamate, as these were seen to be measured individually.

Figure 2A illustrates, which parameters are ubiquitously controlled for all experiments: agitation rate, bioreactor type, cell type, media formulation, microcarrier type, seeding density, and temperature are controlled in every article analysed and should thus be considered CPPs (28–78, 78, 79). Additionally, percentage of CO_2_ in the gaseous mixture is highly mentioned and should be considered a CPP. These parameters must be controlled for every cultivation or experiment to allow for a minimum of standardisation of the operation procedure and repeatability of the experiments. Thus, more consideration must be given to those parameters less frequently measured or controlled. Several process parameters determining the nutrient supply, such as glucose and amino acids, and the presence of inhibitory metabolites, such as lactate and ammonia, were identified in the literature review as seen in Figure 2A (Carmelo et al., 2015; Chen et al., 2015; Cunha et al., 2017; de Da Sá Silva et al., 2020; Dos Santos et al., 2014; Eibes et al., 2010; Elseberg et al., 2012; Gadelorge et al., 2018; Gao et al., 2023; Gonzalez Gil et al., 2020; Grein et al., 2016; Jorgenson et al., 2018; Lawson et al., 2017; Kehoe et al., 2012; Lam et al., 2017; Leber et al., 2017; López-Fernández et al., 2024a; López-Fernández et al., 2024b; Mizukami et al., 2016; Moreira et al., 2020; Noronha et al., 2021; Osiecki et al., 2015; Petry et al., 2016; Rafiq et al., 2016; Rafiq et al., 2013; Rotondi et al., 2021; Santos et al., 2011; Sart et al., 2010; Schirmai et al., 2014; Sion et al., 2020; Sousa et al., 2015; Sunil et al., 2013; Tozetti et al., 2017; Yu et al., 2009; Yuan et al., 2014; Zhang et al., 2022; Zhang et al., 2023; Zhou et al., 2013). Additionally, physicochemical properties of the media, such as the amount of dissolved oxygen (DO), pH, aeration, osmolality, and permittivity, were identified as seen in Figure 2A. Some of these parameters are interdependent, as most media are buffered using a carbonate system; the amount of CO_2_ in the gas supply is used to control pH, and headspace aeration is key to control DO in the media. Establishing metrologies for all process parameters given in Figure 2A is time-consuming and costly, so a prioritisation must be made. In Figure 2B the above-mentioned ubiquitous process parameters are excluded and a weighted Pareto analysis is applied to the remaining parameters considering criticality and cost of implementation for each process parameter. The weighted Pareto analysis identifies glucose and lactate concentration, headspace aeration, DO and pH as CPPs. The concentration of amino acids, glutamine, and glutamate, as well as the shear stress or osmolality in the bioreactor, are less often considered in comparison.

As glucose is the main substrate in most MSC cultivations, its levels are known to impact proliferation and viability of MSCs (Nosrati et al., 2024). Especially in combination with hypoxic conditions, it also influences differentiation capacity, mitochondrial activity and apoptosis (Lau et al., 2020; Almahasneh et al., 2024). Similarly, lactate levels have been shown to decrease proliferation, modulate gene expression and stemness of MSCs (Zieker et al., 2008; Schneider et al., 2012; Sun et al., 2020) stressing the criticality of glucose and lactate levels for MSC expansion. In addition, culturing MSCs from different cell sources in normoxic and hypoxic conditions has been shown to impact cell proliferation, modulate the differentiation capability and expression of pluripotency factors and influence the immunomodulatory capacity of the secretome of MSCs (Lavrentieva et al., 2020; Obradovic et al., 2019; Pezzi et al., 2017; Xu et al., 2022). Concerning the influence of pH, acidic cultivation conditions decrease expansion and viability of MSCs as well as modulate chemokine expression, while alkaline pH might improve differentiation of MSCs into osteoblasts (Wuertz et al., 2009; Bischoff et al., 2008; Fliefel et al., 2016). To ensure high-quality manufacturing of MSCs, these parameters should be considered as CPPs in addition to the ubiquitous measured parameters discussed above.

## Discussion

The expansion of MSCs in bioreactor systems becomes increasingly relevant as more therapies transition through clinical trials. With increased throughput, QbD approaches are being applied to these therapies to ensure consistent, high-quality manufacturing of therapies. While some aspects of MSC manufacturing, such as the extraction of cells and cultivation in plate or flask-based cultures, have been reviewed in detail, the specifics of bioreactor-based MSC expansion were reviewed in this article. A survey of quality attributes and process parameters of MSCs expanded in bioreactor systems was conducted, and a list of quality attributes and process parameters compiled. For the quality parameters, findings of previous studies were confirmed with cell count and viability for dosage, immunophenotype for identity, and trilinear differentiation for potency being identified as CQAs. However, it has been discussed additional parameters relating to the potency of the MSCs depending on the application and additional parameters relating to safety, regarding regulatory guidelines, must be considered in addition. For process parameters, several parameters were identified as being ubiquitously controlled or monitored in bioreactor-based expansion of MSCs: agitation rate, bioreactor type, cell type, media formulation, microcarrier type, seeding density, and temperature are controlled in every article surveyed and percentage of CO_2_ in the gaseous mixture highly frequently. To improve consistency of quality, the process monitoring should be expanded to include more parameters that are not only monitored but also controlled. For prioritisation, a weighted Pareto Analysis was applied to the remaining process parameters and glucose and Lactate concentration, DO, headspace aeration and pH in the media were additionally classified as CPPs. A prerequisite for improved process not discussed so far is the availability of process analytical technology (PAT). As a direct process control is only feasible with in-line sensors for all relevant parameters integrated in the bioreactor system and connected to variables which can be manipulated. While DO and pH are often measured in-line, metabolite concentration is less frequently integrated in-line, but rather measured off-line or at-line. More sophisticated PAT based on enzymatic or spectroscopic principles is not yet frequently integrated in MSC manufacturing. This severely limits the degree of process control currently in use for these therapies and the achievable process consistency. More sophisticated PAT would allow for a detailed investigation of the influence that different levels of process parameters have on the quality of cells, which would be the basis for any process control established and improving consistency and quality of MSC therapy manufacturing.
