# Supplementary material for: Identification of critical process parameters and quality attributes for bioreactor-based expansion of human MSCs
Source: Front Bioeng Biotechnol. 2025 Aug 21;13:1608194. doi: 10.3389/fbioe.2025.1608194 (PMC12408662; doi:10.3389/fbioe.2025.1608194)
Supplement: Supplementary file 1 [file DataSheet2.pdf]

names	Weighting: Costs		Weighting: Risks		Frequency
growth rate (µ)	5	calculation, no additional investment required	2	criticality dependnet on process and product design, not widely reported in literature as a CQA or by regulatory documention	40
Adipogenic Diff.	4	Kits available (<5000 €), labour intensive to conduct	4	Critical to differentiation capability of MSCs, previously identified in literature as CQA across applications	53
Apoptosis	3	Kits avialble (<5000 €), often combined with flow cytometry (high costs/standard analytics)	2	criticality dependnet on process and product design, not widely reported in literature as a CQA or by regulatory documention	5
CD105	3	invest necessary (>100000€), commonly flow cytometry available in cell culture laboratories	4	Likely critical to quality in terms of identity, positive MSC marker widely used	57
CD106	3	invest necessary (>100000€), commonly flow cytometry available in cell culture laboratories	2	adiditional biomarker, not widely used, depending on cell origin and specific subfunction	5
CD11b	3	invest necessary (>100000€), commonly flow cytometry available in cell culture laboratories	3	additional negative MSC marker, used in addition to other markers, depending on cell origin	9
CD13	3	invest necessary (>100000€), commonly flow cytometry available in cell culture laboratories	2	adiditional biomarker, not widely used, depending on cell origin and specific subfunction	4
CD14	3	invest necessary (>100000€), commonly flow cytometry available in cell culture laboratories	3	additional negative MSC marker, used in addition to other markers, depending on cell origin	23
CD140b	3	invest necessary (>100000€), commonly flow cytometry available in cell culture laboratories	3	potentially positive MSC marker, may be used in addition to common markers	1
CD144	3	invest necessary (>100000€), commonly flow cytometry available in cell culture laboratories	2	adiditional biomarker, not widely used, depending on cell origin and specific subfunction	1

CD146	3	invest necessary (>100000€), commonly flow cytometry available in cell culture laboratories	2	adiditional biomarker, not widely used, depending on cell origin and specific subfunction	7
CD157	3	invest necessary (>100000€), commonly flow cytometry available in cell culture laboratories	2	adiditional biomarker, not widely used, depending on cell origin and specific subfunction	1
CD166	3	invest necessary (>100000€), commonly flow cytometry available in cell culture laboratories	3	potentially positive MSC marker, may be used in addition to common markers	6
CD19	3	invest necessary (>100000€), commonly flow cytometry available in cell culture laboratories	3	additional negative MSC marker, used in addition to other markers, depending on cell origin	22
CD200	3	invest necessary (>100000€), commonly flow cytometry available in cell culture laboratories	2	adiditional biomarker, not widely used, depending on cell origin and specific subfunction	1
CD271	3	invest necessary (>100000€), commonly flow cytometry available in cell culture laboratories	3	potentially positive MSC marker, may be used in addition to common markers	1
CD274	3	invest necessary (>100000€), commonly flow cytometry available in cell culture laboratories	2	adiditional biomarker, not widely used, depending on cell origin and specific subfunction	2
CD29	3	invest necessary (>100000€), commonly flow cytometry available in cell culture laboratories	3	potentially positive MSC marker, may be used in addition to common markers	10
CD31	3	invest necessary (>100000€), commonly flow cytometry available in cell culture laboratories	3	additional negative MSC marker, used in addition to other markers, depending on cell origin	17
CD34	3	invest necessary (>100000€), commonly flow cytometry available in cell culture laboratories	3	additional negative MSC marker, used in addition to other markers, depending on cell origin	45
CD44	3	invest necessary (>100000€), commonly flow cytometry available in cell culture laboratories	3	potentially positive MSC marker, may be used in addition to common markers	16

CD45	3	invest necessary (>100000€), commonly flow cytometry available in cell culture laboratories	4	negative marker for MSCs widely used	40
CD49e	3	invest necessary (>100000€), commonly flow cytometry available in cell culture laboratories	2	adiditional biomarker, not widely used, depending on cell origin and specific subfunction	2
CD54	3	invest necessary (>100000€), commonly flow cytometry available in cell culture laboratories	2	adiditional biomarker, not widely used, depending on cell origin and specific subfunction	1
CD70	3	invest necessary (>100000€), commonly flow cytometry available in cell culture laboratories	2	adiditional biomarker, not widely used, depending on cell origin and specific subfunction	2
CD71	3	invest necessary (>100000€), commonly flow cytometry available in cell culture laboratories	2	adiditional biomarker, not widely used, depending on cell origin and specific subfunction	1
CD73	3	invest necessary (>100000€), commonly flow cytometry available in cell culture laboratories	4	Likely critical to quality in terms of identity, positive MSC marker widely used	56
CD79a	3	invest necessary (>100000€), commonly flow cytometry available in cell culture laboratories	2	adiditional biomarker, not widely used, depending on cell origin and specific subfunction	1
CD80	3	invest necessary (>100000€), commonly flow cytometry available in cell culture laboratories	2	adiditional biomarker, not widely used, depending on cell origin and specific subfunction	8
CD90	3	invest necessary (>100000€), commonly flow cytometry available in cell culture laboratories	4	Likely critical to quality in terms of identity, positive MSC marker widely used	55
Cell Count	4	manual (<1000 €), or invest in automated cell counters (>100000€)	5	Critical to Dose, dose one of the main attributes mentioned in regulatory documents across all medicinal products	76
Cell Migration	4	Commercial Kits available (>5000 €)	2	criticality dependnet on process and product design, not widely reported in literature as a CQA or by regulatory documention	1

CFU Assay	5	Standard Cell Cultivation methods and staining	2	criticality dependnet on process and product design, not widely reported in literature as a CQA or by regulatory documention	8
Chondrogenic Diff.	4	Kits available (<5000 €), labour intensive to conduct	4	Critical to differentiation capability of MSCs, previously identified in literature as CQA across applications	51
Karyotyping/Cytogenic Analysis	4	External services can be contracted (>5000), use of standard cell culture mthods, chemical treatments/staining	5	CQA for Safety, pertaining to cell age and tumorgeneicty	10
Cytokine Analysis	5	Commercial Kits (i.e. ELISA) availble (>5000 €)	3	potentially critical to potency of MSCs, discussed as additional potency assay	5
death Rate	5	calculation, no additional investment required	2	criticality dependnet on process and product design, not widely reported in literature as a CQA or by regulatory documention	2
doubling Time	5	calculation, no additional investment required	2	criticality dependnet on process and product design, not widely reported in literature as a CQA or by regulatory documention	13
Population Doubling	5	calculation, no additional investment required	4	Likely critical to cell age of cells, mentioned as a CQA previously	12
Expansion Factor	5	calculation, no additional investment required	2	criticality dependnet on process and product design, not widely reported in literature as a CQA or by regulatory documention	9
Fold Increase	5	calculation, no additional investment required	2	criticality dependnet on process and product design, not widely reported in literature as a CQA or by regulatory documention	30
RNA analysis/Transcriptomics (Microarrays or RNA seq)	3	can be externally contracted (>5000 €), otherwise investmenbt required (>100000€)	3	potentially critical to potency of MSCs, discussed as additional potency assay	8

HLA-ABC	3	invest necessary (>100000€), commonly flow cytometry available in cell culture laboratories	2	adiditional biomarker, not widely used, depending on cell origin and specific subfunction	3
HLA-DR	3	invest necessary (>100000€), commonly flow cytometry available in cell culture laboratories	2	additional negative MSC marker, used in addition to other markers, depending on cell origin	38
HUVEC Migration / tube formation	5	Standard Cell culture methods, no additional investment requried	2	criticality dependnet on process and product design, not widely reported in literature as a CQA or by regulatory documention	1
IDO formation	1	requires UPLC system, high investment required (>250000€)	2	criticality dependnet on process and product design, not widely reported in literature as a CQA or by regulatory documention	2
IFNy assay	4	Commercial Kits available (>5000 €)	2	criticality dependnet on process and product design, not widely reported in literature as a CQA or by regulatory documention	2
(Immuno)fluorescene Microscopy	3	Investment necessary (>100000€), commonly available in cell culture laboratories	2	criticality dependnet on process and product design, not widely reported in literature as a CQA or by regulatory documention	21
Metabolic Activity	3	Commercial Kits available (>5000 €), requires plate reader (<50000 €), which is commonly available in cell culture laboratories	2	criticality dependnet on process and product design, not widely reported in literature as a CQA or by regulatory documention	3
Metoblomics (MS/MS)	1	high invest (>250000€) necessary, labour intensive	2	criticality dependnet on process and product design, not widely reported in literature as a CQA or by regulatory documention	2
Animal model	1	high costs (>250000€), specific costs depend on type and number of animals used	1	Used to elucidate quality/effifacy of cell product, unsuited as a quality attribute	5

Osteogenic Diff.	4	Kits available (<5000 €), labour intensive to conduct	4	Critical to differentiation capability of MSCs, previously identified in literature as CQA across applications	56
ParticleDiameter / Aggregation	4	Determined using standard microscopy, available as a function in some automated cell counters	2	criticality dependnet on process and product design, not widely reported in literature as a CQA or by regulatory documention	7
Plastic Adherence	5	standard cell culture cultivation procedure, no additional costs	4	Critical to identity of MSCs	9
Proteomics	1	high invest (>250000€) necessary, labour intensive	2	criticality dependnet on process and product design, not widely reported in literature as a CQA or by regulatory documention	2
RT-PCR or qPCR	3	some invest necessary, depending on specialisation of equipment (30000 - 100000 €)	3	potentially critical to potency of MSCs, discussed as additional potency assay	14
Secretome Analysis	3	Strongly dependent on method used, some ELISA kits available (>5000 €), requires reader, externally contratced arrays available (>15000 €)	3	potentially critical to potency of MSCs, discussed as additional potency assay	10
Spec. Glc Consumption	5	calculation, no additional investment required	2	criticality dependnet on process and product design, not widely reported in literature as a CQA or by regulatory documention	24
Spec. Lac Production	5	calculation, no additional investment required	2	criticality dependnet on process and product design, not widely reported in literature as a CQA or by regulatory documention	24
Telomer / Telomerase	3	Commercial ELISA Kits available (>5000 €), plate reader requried	5	CQA for Safety, pertaining to cell age and tumorgeneicty	3
T Lymphocyte Proliferation	3	Standard Cell Culture cultivation methods, PBMCs/T Cell isolation and albelling required (Kits available >5000€), flow cytometer requried	3	potentially critical to potency of MSCs, discussed as additional potency assay	7

Viability	4	manual (<1000 €), or invest in automated cell counters (>100000€)	5	Critical to Dose, dose one of the main attributes mentioned in regulatory documents across all medicinal products	76
Yield (Lactate/Glucose)	5	calculation, no additional investment required	2	criticality dependnet on process and product design, not widely reported in literature as a CQA or by regulatory documention	23
Senescence	3	Commercial b-Galactosidase Kits available (>5000€), inverted microscope requried	2	criticality dependnet on process and product design, not widely reported in literature as a CQA or by regulatory documention	4
PBMC proliferation	3	Standard cell culture mthods, PBMCs required, beads/kits required (>5000€), reader required	3	potentially critical to potency of MSCs, discussed as additional potency assay	4
Cell Size	4	Determined using standard microscopy, available as a function in some automated cell counters	2	criticality dependnet on process and product design, not widely reported in literature as a CQA or by regulatory documention	4
Scanning Electron Microscopy	1	high invest (>250000€) necessary	1	Likely unsuited as critical quality attribute	1
Short Tandem Repeats analysis	4	Contracted externally (>5000€)	5	CQA for Safety, pertaining to cell age and tumorgeneicty	1
F-actin staining	3	Staining procedure, requires fluoresence microscopy	2	criticality dependnet on process and product design, not widely reported in literature as a CQA or by regulatory documention	1
Histology	2	Complex procedure, usually contracted externally (>5000€), otherwise very high investment	2	criticality dependnet on process and product design, not widely reported in literature as a CQA or by regulatory documention	2

Cell Productivity	5	calculation, no additional investment required	2	criticality dependnet on process and product design, not widely reported in literature as a CQA or by regulatory documention	1
Endotoxin	3	some invest necessary (ca. 30000€)	5	CQA for Safety, mentioned by EMA and FDA as part of regulatory documentation	2
Sterility/Microbial agents	2	invest necessary (>100000€), can be externally contracted	5	CQA for Safety, mentioned by EMA and FDA as part of regulatory documentation	2
Mycoplasma	3	detected via qPCR, some invest necessary, depending on specialisation of equipment (30000 - 100000 €), can be externally contracted	5	CQA for Safety, mentioned by EMA and FDA as part of regulatory documentation	2

5	low cost / no additional hardware required	5	known CQA of MSCs across all applications, i.e. attributes pertaining to safety; explicitly mentioned in regulatory documents
4	Kits or standard multi-use probes available, based on calculations/models (<10000€)	4	attribute frequently listed as a critical quality attribute for MSCs, i.e. identity markers
3	some invest necessary, potentially measureable for <100.000 €/ higher invest, but considered standard analytical method	3	attribute frequently discussed as a poetntially critical quality attribute of MSCs
2	invest necessary (>100.000€)	2	criticality dependnet on process and product design, not widely reported in literature as a CQA or by regulatory documention
1	high invest necessary (>250000€)	1	attribute not critical to quality or unsuited as a quality attribute
