# Supplementary material for: Identification of critical process parameters and quality attributes for bioreactor-based expansion of human MSCs
Source: Front Bioeng Biotechnol. 2025 Aug 21;13:1608194. doi: 10.3389/fbioe.2025.1608194 (PMC12408662; doi:10.3389/fbioe.2025.1608194)

S2: PRISMA diagram for literature review

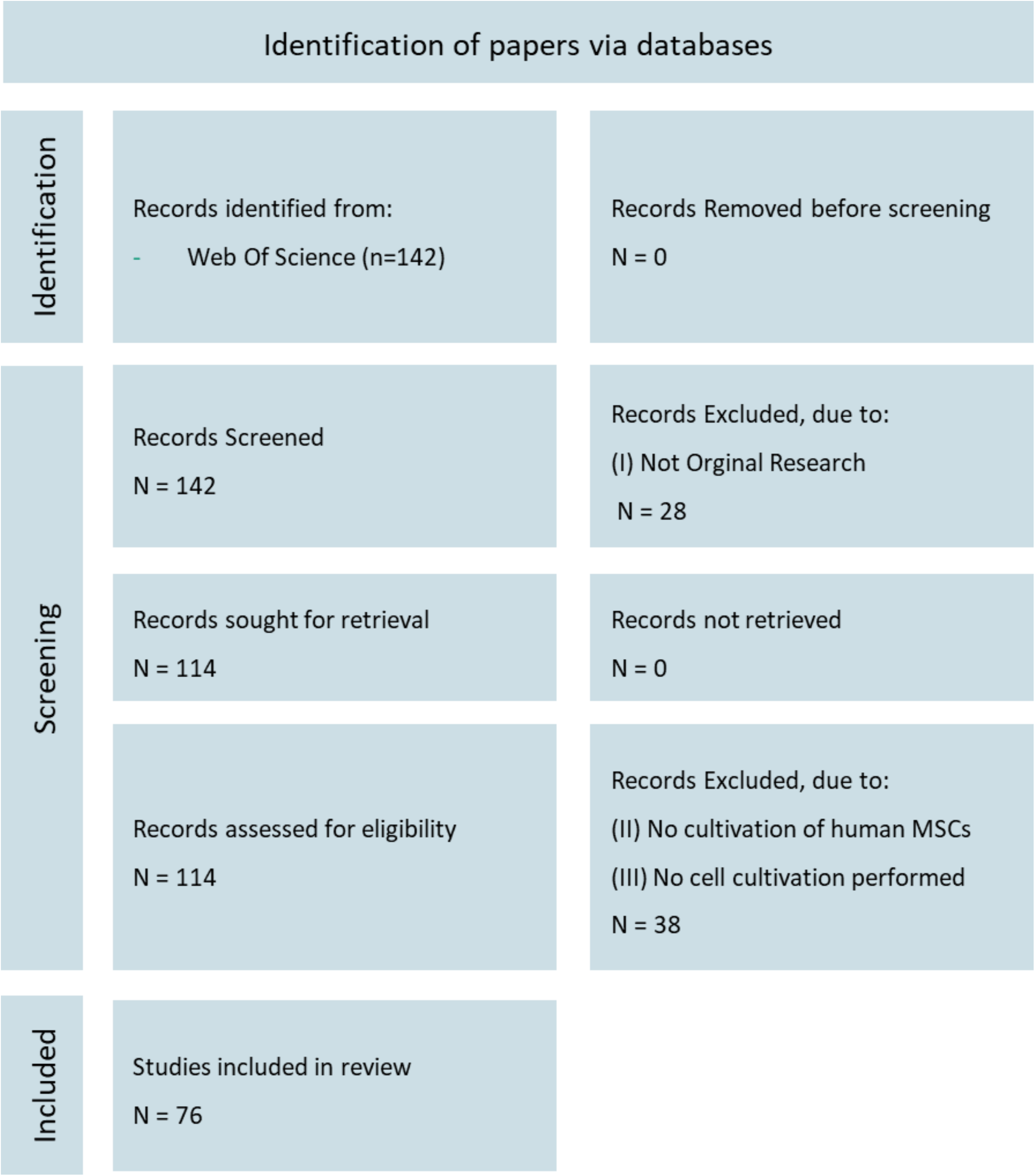

S5: Weighted Pareto Analysis

From the articles considered, process parameters and quality attributes measured are recorded for each article. Any attribute of the cells is considered a quality attribute, whether measured throughout the process (in-process quality control) or in the final product (end-product quality control). Parameters calculated from these attributes, i.e. growth rate, are also considered as quality attributes of the MSCs. Any process parameter monitored was considered a “process parameter” and no distinction was made between monitored or controlled parameters; both types

were recorded equally. In total, 79 different quality attributes and 41 process parameters were identified in the articles reviewed and the number of occurrences (N_{Occ}) recorded across the articles. Additionally, a weighting in terms of cost of implementation ($weight_{costs}$; 5: low cost – 1: high invest necessary) as well as criticality to product or to process stability ($weight_{criticality}$; 5: known to be critical to quality – 1: not critical to quality) is applied. The number of occurrences and weightings with reasoning are given in S3 and S4.

To prioritize parameters and attributes to be measured and use available resources effectively, a weighted Pareto analysis is applied. Weighted counts are calculated according to (1), sorted in descending order and the cumulative percentage is calculated. In line with the Pareto principle, a threshold is set at 80 % of the cumulative percentage to identify the parameters most likely to be critical.

$$weighted\ counts = (N_{Occ} * Weight_{costs} + N_{Occ} * Weight_{criticality}) \quad (1)$$

S4: PRISMA diagram for literature review

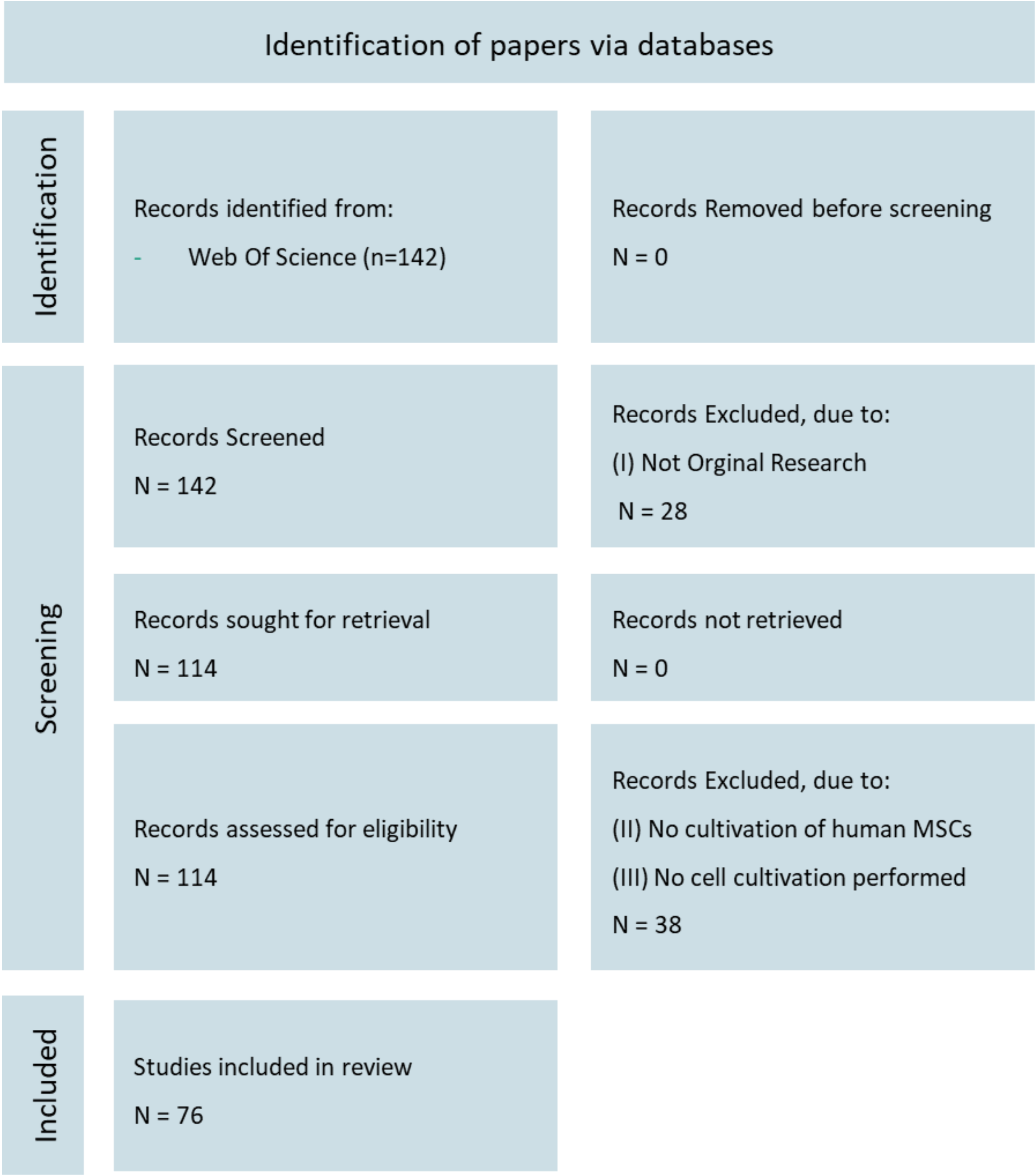

Supplement: Supplementary file 2 [file DataSheet4.pdf]
