# Supplementary material for: Identification of critical process parameters and quality attributes for bioreactor-based expansion of human MSCs
Source: Front Bioeng Biotechnol. 2025 Aug 21;13:1608194. doi: 10.3389/fbioe.2025.1608194 (PMC12408662; doi:10.3389/fbioe.2025.1608194)
Supplement: Supplementary file 3 [file DataSheet3.pdf]

names	Weighting: Costs		Weighting Risks		Frequency
%CO2	5	present in most incubation systems, no additional hardware required	4	controls media pH, risk of pH shift	62
DAPI staining	4	Specialised Kits (<1000€) available	2	verifies cell attachement, risk of missing cell attachement (unlikely after process development)	12
Amino Acid	2	Invest necessary (> 100.000€), complex analytical methods required (i.e. spectroscopy, HPLC)	2	controlled by media, low risk of secondary substrate limitation	1
Ammonia	3	Probes (Spectroscopy, ca. 80000 €) available, bioanalyzers (>100.000€) often available in standard laboratories	2	controlled by media, risk of product inhibition	25
Adipose Derived MSCs	5	costs dependent on the application/researching entity	5	must be controlled for process set-up / screening	14
Bone Marrow MSCs	5	costs dependent on the application/researching entity	5	must be controlled for process set-up / screening	27
Ear MSCs	5	costs dependent on the application/researching entity	5	must be controlled for process set-up / screening	2
Fetal MSCs	5	costs dependent on the application/researching entity	5	must be controlled for process set-up / screening	3
Early MSCs	5	costs dependent on the application/researching entity	5	must be controlled for process set-up / screening	4
Placental MSCs	5	costs dependent on the application/researching entity	5	must be controlled for process set-up / screening	3
Synovial Fluid MSCs	5	costs dependent on the application/researching entity	5	must be controlled for process set-up / screening	2
Umbilical Cord MSCs	5	costs dependent on the application/researching entity	5	must be controlled for process set-up / screening	17
Wharton-Jelly MSCs	5	costs dependent on the application/researching entity	5	must be controlled for process set-up / screening	9
Embryonic stem cell MSCs	5	costs dependent on the application/researching entity	5	must be controlled for process set-up / screening	1

Dissolved Oxygen	4	Multi-Use probes (ca. 1000 €) readily available	4	monitors level of available oxygen, in combination with agitation/headspace aeration may control levels of available	39
Glucose	4	Probes (Spectroscopy ca. 80000€, single-use enzymatic probes (ca. 300 €) available, bioanalyzers (>100.000€) often available in standard laboratories	3	controlled by frequency of media exchange, risk of main substrate limitation	52
Glutamine	3	Probes (Spectroscopy ca. 80000€, bioanalyzers (>100.000€) available	3	controlled by frequency of media exchange, risk of secondary substrate limitation	18
Glutamate	3	Probes (Spectroscopy ca. 80000€, bioanalyzers (>100.000€) available	3	controlled by frequency of media exchange, risk of secondary substrate limitation	6
Headspace aeration	5	Readily available for systems requiring aeration, no additioanl hardware required	4	controls oxygen supply to cells, more critical in non-normoxic conditions	35
Lactate	4	Probes (Spectroscopy ca. 80000€, enzymatic probes (ca. 300 €) available, bioanalyzers (>100.000€) often available in standard laboratories	3	controlled by media, risk of product inhibition	50
Microcarrier	5	depending on specific formulation, prerequisite for any experiment	5	must be controlled for process set-up / screening	74
Osmolality	2	Invest necessary, bioanalyzers (>100.000€) available	2	verifies osmolality in media, mainly controlled by media composition, low risk to cultivation performance	1
Permitivity	4	Probes commcerically available (ca. 1500€)	4	permittivity may indicate viability of cells in bioreactor system, viability is critical to quality	1
pH	4	Multi-Use probes (ca. 500 €) readily available	4	verifies correct pH, risk of pH shift	39
Agitation	5	Readily available for systems requiring agitation, no additioanl hardware required	5	controls agitation rate, risk of too high or low agitation, shear stress or inhomogeneities	75
Seeding Density	5	Costs mostly affected by cells chosen	5	must be controlled for process set-up /	76
Media Formulation	5	depending on specific formulation, prerequisite for any experiment	5	must be controlled for process set-up / screening	76
Shear Stress	4	based on labour intensive calculations and models, no invest necessary	3	risk of to high shear stress might disrupt cultivation, directly connected to agitation and aeration	7
Spinner Flask Bioreactor	4	low invest (10000€)standard incubator with rollers, low-cost single use vessels available (<500 €)	5	must be controlled for process set-up / screening	50

Stirred Tank Bioreactor	3	invest necessary (50000-150000€), multiple-use vessels available, single-use vessels (500 - 1500€/cultivation)	5	must be controlled for process set-up / screening	40
Temperature	5	present in incubation systems/ bioreactors; no additional hardware required	4	must be controlled for good cultivation outcome	76
Taylor Vortex Bioreactor	2	invest necessary (50000 - 150000€, single-use vessels (500 - 1500€/cultivation) with specialised equipment	5	must be controlled for process set-up / screening	1
Vertical Wheel Bioreactor	2	invest necessary (50000 - 150000€, single-use vessels (500 - 1500€/cultivation) with specialised equipment	5	must be controlled for process set-up / screening	1
WAVE Bioreactor	2	invest necessary (50000 - 150000€, single-use vessels (500 - 1500€/cultivation) with specialised equipment	5	must be controlled for process set-up / screening	4
Packed Bed Bioreactor	2	invest necessary (50000 - 150000€, single-use vessels (500 - 1500€/cultivation) with specialised equipment	5	must be controlled for process set-up / screening	1
Flow Rate	5	present in perfusion bioreactor systems, no additional hardware required	5	must be controlled for process set-up / screening in any perfusion experiment, otherwise irrelevant	2
Shake Flask	4	low invest (10000€),standard shaking incubators, low cost single-use vessels available (<500 €)	5	must be controlled for process set-up / screening	1
TotalProteinContent	2	invest necessary, bioanalyzers (>100.000€) available	2	verifies total protein content	1
LactateDehydrogenase	2	invest necessary, bioanalyzers (>100.000€) available	1	verifies activity of lactate dehydrogenase, interesting to monitor	1
Potassium	2	invest necessary, bioanalyzers (>100.000€) available	1	verifies potassium levels, mostly controlled by media composition, very low risk to cultivation outcome	1

5	low cost / no additional hardware required/costs covered by other invests listed; prerequisite for cultivation/depending on specific choice	5	must be controlled for any process set-up for a minimal level of process control
4	Kits or standard multi-use probes available, based on calculations/models (<10000€)	4	high risk of main cultivation parameter deviating without controlling this parameter
3	some invest necessary, potentially measureable for <100.000 €, multi-use equipment available	3	risk of detrimental cultivation outcome if this parameter is not monitored
2	invest necessary (>100.000€), only single-use equipment available	2	interesting parameter to monitor to verify/investigate cultivation performance, lower risk to cultivation performance
1	high invest necessary (>250000€)	1	interesting parameter to monitor to verify/investigate cultivation performance, very low risk to cultivation performance
