# Supplementary material for: Identification of critical process parameters and quality attributes for bioreactor-based expansion of human MSCs
Source: Front Bioeng Biotechnol. 2025 Aug 21;13:1608194. doi: 10.3389/fbioe.2025.1608194 (PMC12408662; doi:10.3389/fbioe.2025.1608194)
Supplement: Supplementary file 4 [file DataSheet1.pdf]

No.	Authors	Article Title	Type	Year	DOI	Exclusion criteria
1	Yu et al	Ex Vitro Expansion of Human Placenta-Derived Mesenchymal Stem Cells in Stirred Bioreactor	Article	2009	10.1007/s12010-009-8556-5	included
2	Zhou et al	Ex vivo expansion of bone marrow mesenchymal stem cells using microcarrier beads in a stirred bioreactor	Article	2013	10.1007/s12257-012-0512-5	included
3	Mizukami et al	Stirred tank bioreactor culture combined with serum-/xenogeneic-free culture medium enables an efficient expansion of umbilical cord-derived mesenchymal stem/stromal cells	Article	2016	10.1002/biot.201500532	included
4	Rafiq et al	Culture of human mesenchymal stem cells on microcarriers in a 5 l stirred-tank bioreactor	Article	2013	10.1007/s10529-013-1211-9	included
5	Schirmaier et al	Scale-up of adipose tissue-derived mesenchymal stem cell production in stirred single-use bioreactors under low-serum conditions	Article	2014	10.1002/elsc.201300134	included
6	Grein et al	Multiphase mixing characteristics in a microcarrier-based stirred tank bioreactor suitable for human mesenchymal stem cell expansion	Article	2016	10.1016/j.procbio.2016.05.010	included
7	Noronha et al	Hypoxia priming improves in vitro angiogenic properties of umbilical cord derived-mesenchymal stromal cells expanded in stirred-tank bioreactor	Article	2021	10.1016/j.bej.2021.107949	included

8	dos Santos et al	A Xenogeneic-Free Bioreactor System for the Clinical-Scale Expansion of Human Mesenchymal Stem/Stromal Cells	Article	2014	10.1002/bit.25187	included
9	Sart et al	Modulation of mesenchymal stem cell actin organization on conventional microcarriers for proliferation and differentiation in stirred bioreactors	Article	2013	10.1002/term.545	included
10	Lawson et al	Process development for expansion of human mesenchymal stromal cells in a 50 L single-use stirred tank bioreactor	Article	2017	10.1016/j.bej.2016.11.020	included
11	Mizukami et al	Proteomic Identification and Time-	Article	2019	10.3389/fbioe.2019.00154	included
12	da Silva et al	Mesenchymal stromal cells maintain the major quality attributes when expanded in different bioreactor systems	Article	2020	10.1016/j.bej.2020.107693	included
13	Zhang et al	Large-Scale Expansion of Human Umbilical Cord-Derived Mesenchymal Stem Cells in a Stirred Suspension Bioreactor Enabled by Computational Fluid Dynamics Modeling	Article	2022	10.3390/bioengineering9070274	included
14	Kurogi et al	Study on the Umbilical Cord-Mesenchymal Stem Cell Manufacturing Using Clinical-Grade Culture Medium	Article	2022	10.1089/ten.tec.2021.0207	included
15	Yuan et al	Improved expansion of human	Article	2014	10.1002/term.1515	included
16	Eibes et al	Maximizing the ex vivo expansion of human mesenchymal stem cells using a microcarrier-based stirred culture system	Article	2010	10.1016/j.jbiotec.2010.02.015	included

17	Moreira et al	Successful Use of Human AB Serum to Support the Expansion of Adipose Tissue-Derived Mesenchymal Stem/Stromal Cell in a Microcarrier-Based Platform	Article	2020	10.3389/fbioe.2020.00307	included
18	Timmins et al	Closed system isolation and scalable expansion of human placental mesenchymal stem cells	Article	2012	10.1002/bit.24425	included
19	Sart et al	Ear mesenchymal stem cells: An efficient adult multipotent cell population fit for rapid and scalable expansion	Article	2009	10.1016/j.jbiotec.2008.12.011	included
20	Carmelo et al	A xeno-free microcarrier-based stirred culture system for the scalable expansion of human mesenchymal stem/stromal cells isolated from bone marrow and adipose tissue	Article	2015	10.1002/biot.201400586	included
21	Kehoe et al	Scale-up of Human Mesenchymal Stem Cells on Microcarriers in Suspension in a Single-use Bioreactor	Article	2012		included
22	Sart et al	Influence of culture parameters on ear mesenchymal stem cells expanded on microcarriers	Article	2010	10.1016/j.jbiotec.2010.08.003	included
23	Jorgenson et al	Production of Adult Human Synovial Fluid-Derived Mesenchymal Stem Cells in Stirred-Suspension Culture	Article	2018	10.1155/2018/8431053	included

24	Sousa et al	Production of Oncolytic Adenovirus and Human Mesenchymal Stem Cells in a Single-Use, Vertical-Wheel Bioreactor System: Impact of Bioreactor Design on Performance of Microcarrier-Based Cell Culture Processes	Article	2015	10.1002/btpr.2158	included
25	Chung et al	Fabrication of Adipose-Derived Mesenchymal Stem Cell Aggregates using Biodegradable Porous Microspheres for Injectable Adipose Tissue Regeneration	Article	2011	10.1163/092050609X12580983495681	included
26	Gil et al	Feasibility of the taylor vortex flow bioreactor for mesenchymal stromal cell expansion on microcarriers	Article	2020	10.1016/j.bej.2020.107710	included
27	Nienow et al	Mixing theory for culture and harvest in bioreactors of human mesenchymal stem cells on microcarriers	Article	2016	10.1134/S0040579516060117	included
28	Rafiq et al	Systematic microcarrier screening and agitated culture conditions improves human mesenchymal stem cell yield in bioreactors	Article	2016	10.1002/biot.201400862	included
29	Tozetti et al	Expansion Strategies for Human Mesenchymal Stromal Cells Culture under Xeno-Free Conditions	Article	2017	10.1002/btpr.2494	included
30	Leber et al	Microcarrier choice and bead-to-bead transfer for human mesenchymal stem cells in serum-containing and chemically defined media	Article	2017	10.1016/j.procbio.2017.03.017	included

31	Sion et al	A new perfusion mode of culture for WJ-MSCs expansion in a stirred and online monitored bioreactor	Article	2021	10.1002/bit.27914	included
32	Cunha et al	Bioprocess integration for human mesenchymal stem cells: From up to downstream processing scale-up to cell proteome characterization	Article	2017	10.1016/j.jbiotec.2017.01.014	included
33	Rotondi et al	Design and development of a new ambr250 (R) bioreactor vessel for improved cell and gene therapy applications	Article	2021	10.1007/s10529-021-03076-3	included
34	Jing et al	Growth Kinetics of Human Mesenchymal Stem Cells in a 3-L Single-Use, Stirred-Tank Bioreactor	Article	2013		included
35	Yan et al	Dispersible and Dissolvable Porous Microcarrier Tablets Enable Efficient Large-Scale Human Mesenchymal Stem Cell Expansion	Article	2020	10.1089/ten.tec.2020.0039	included
36	Kurogi et aol	Umbilical cord derived mesenchymal stromal cells in microcarrier based industrial scale culture sustain the immune regulatory functions	Article	2021	10.1002/biot.202000558	included
37	Gadelorge et al	Clinical-scale expansion of adipose-derived stromal cells starting from stromal vascular fraction in a single-use bioreactor: proof of concept for autologous applications	Article	2018	10.1002/term.2377	included

38	Chen et al	Increasing efficiency of human mesenchymal stromal cell culture by optimization of microcarrier concentration and design of medium feed	Article	2015	10.1016/j.jcyt.2014.08.011	included
39	Lam et al	Biodegradable poly-epsilon-caprolactone microcarriers for efficient production of human mesenchymal stromal cells and secreted cytokines in batch and fed-batch bioreactors	Article	2017	10.1016/j.jcyt.2016.11.009	included
40	Sion et al	Effects of microcarriers addition and mixing on WJ-MSC culture in bioreactors	Article	2020	10.1016/j.bej.2020.107521	included
41	Petry et al	Cord Mesenchymal Stromal Cells	Article	2016	10.1155/2016/4834616	included
42	dos Santos et al	Toward a Clinical-Grade Expansion of Mesenchymal Stem Cells from Human Sources: A Microcarrier-Based Culture System Under Xeno-Free Conditions	Article	2011	10.1089/ten.tec.2011.0255	included
43	Elseberg et al	Microcarrier-based expansion process for hMSCs with high vitality and undifferentiated characteristics	Article	2012	10.5301/ijao.5000077	included
44	Dosta et al	Scale-up manufacturing of gelatin-based microcarriers for cell therapy	Article	2020	10.1002/jbm.b.34624	included
45	Gao, et al	A scalable culture system incorporating microcarrier for specialised mesenchymal stem cells from human embryonic stem cells	Article	2023	10.1016/j.mtbio.2023.100662	included
46	Wang, X et al	Efficient expansion and delayed senescence of hUC-MSCs by microcarrier-bioreactor system	Article	2023	10.1186/s13287-023-03514-1	included

47	Zhang, BT et al	Enhancing mesenchymal stem cells cultivated on microcarriers in spinner flasks via impeller design optimization for aggregated suspensions	Article	2023	10.1186/s40643-023-00707-7	included
48	López-Fernández et	Comparability exercise of critical quality attributes of clinical-grade human mesenchymal stromal cells from the Wharton's jelly: single-use stirred tank bioreactors versus planar culture systems	Article	2024	10.1016/j.jcyt.2023.08.008	included
49	Osiecki et al	Packed Bed Bioreactor for the Isolation and Expansion of Placental-Derived Mesenchymal Stromal Cells	Article	2015	10.1371/journal.pone.0144941	included
50	López-Fernández et	Identification of critical process parameters for expansion of clinical grade human Wharton's jelly-derived mesenchymal stromal cells in stirred-tank bioreactors	Article	2024	10.1002/biot.202300381	included
51	Padhiar et al	GMP compliant clinical grade and xenofree manufacturing of human Wharton?s jelly derived mesenchymal stem cell from pooled donors	Article	2022	10.1016/j.bej.2022.108470	included
52	Kaneko et al	Expansion of human mesenchymal stem cells on poly(vinyl alcohol) microcarriers	Article	2023	10.1016/j.jbiosc.2023.08.003	included
53	Alimperti et al	Serum-Free Spheroid Suspension C	Article	2014	10.1002/btpr.1904	included
54	Bandarra-Tavareset	Dual production of human mesenchy	Article	2024	10.1016/j.jcyt.2024.03.001	included
55	Cao et al	Three-dimensional culture of human	Article	2010	10.1088/1748-6041/5/6/065013	included
56	Costa et al	Enhanced bioprocess control to adva	Article	2023	10.1002/bit.28378	included
57	de Soure et al	Integrated culture platform based on	Article	2017	10.1002/term.2200	included
58	dos Santos et al	Transitioning from static to suspensi	Article	2024	10.1002/btpr.3419	included
59	Fernandes-Platzgum	Optimized operation of a controlled s	Article	2023	10.1002/bit.28449	included

60	Heathman et al	Expansion, harvest and cryopreserva	Article	2015	10.1002/bit.25582	included
61	Heathman et al	Agitation and aeration of stirred-biore	Article	2018	10.1016/j.bej.2018.04.011	included
62	Jossen et al	Theoretical and Practical Issues Tha	Article	2016	10.1155/2016/4760414	included
63	Kaiser et al	Fluid Flow and Cell Proliferation of M	Article	2013	10.1002/cite.201200180	included
64	Lam et al	Multiomics analyses of cytokines, ge	Article	2021	10.1016/j.scr.2021.102272	included
65	Lam et al	Sub-confluent culture of human mes	Article	2019	10.1016/j.jcyt.2019.03.004	included
66	Lin et al	Expansion in microcarrier-spinner cu	Article	2016	10.1016/j.jcyt.2016.03.293	included
67	Ochs et al	Needle to needle robot-assisted mar	Article	2022	10.1002/btm2.10387	included
68	Phelps et al	Extracellular Vesicles Generated by	Article	2024	10.3390/ijms25105219	included
69	Phelps et al	Production of Mesenchymal Progeni	Article	2022	10.1093/stcltm/szab008	included
70	Shekaran et al	Enhanced in vitro osteogenic differer	Article	2015	10.1186/s12896-015-0219-8	included
71	Simao et al	Adipose-derived stem cells (ASCs) c	Article	2023	10.1007/s10529-023-03367-x	included
72	Soder et al	Microcarrier-based clinical-grade ma	Article	2024	10.1016/j.jcyt.2024.07.003	included
73	Tan et al	Serum-free media formulations are c	Article	2015	10.1016/j.jcyt.2015.05.001	included
74	Santhagunam et al	Isolation and ex vivo expansion of sy	Article	2014	10.1016/j.jcyt.2013.10.010	included
75	Shekaran et al	Biodegradable ECM-coated PCL mic	Article	2016	10.1016/j.jcyt.2016.06.016	included
76	Chen et al	Large-Scale Cell Production Based on GMP-Grade Dissolva		2023	10.3791/65469	included
77	Muradoglu et al	Optical stirring in a droplet cell bioreactor	Article	2012	10.1364/BOE.3.002465	no MSC culture, just b
78	Cruz et al	Stirred flow bioreactor modulates chondrocyte growth and extracellular matrix biosynthesis in chitosan scaffolds	Article	2012	10.1002/jbm.a.34174	only chondrocytes, no
79	Wang et al	Myocardial Scaffold-Based Cardiac Tissue Engineering: Application of Coordinated Mechanical and Electrical Stimulations	Article	2013	10.1021/la401702w	no MSCs, cardiac tissu
80	Collignon et al	Large-Eddy Simulations of microcarrier exposure to potentially damaging eddies inside mini-bioreactors	Article	2016	10.1016/j.bej.2015.10.020	Simulation (II)

81	Rebelo et al	Three-dimensional co-culture of human hepatocytes and mesenchymal stem cells: improved functionality in long-term bioreactor cultures	Article	2017	10.1002/term.2099	heptocytes co-cultured
82	Zhang et al	Fabrication of viable and functional pre-vascularized modular bone tissues by coculturing MSCs and HUVECs on microcarriers in spinner flasks	Article	2017	10.1002/biot.201700008	co-culture with other c
83	Orfei et al	Silk/Fibroin Microcarriers for Mesenchymal Stem Cell Delivery: Optimization of Cell Seeding by the Design of Experiment	Article	2018	10.3390/pharmaceutics10040200	only studied microcarri
84	Wyma et al	Non-Newtonian rheology in suspension cell cultures significantly impacts bioreactor shear stress quantification	Article	2018	10.1002/bit.26723	L929 cells, not MSCs
85	Khurshid et al	Osteoarthritic human chondrocytes proliferate in 3D co-culture with mesenchymal stem cells in suspension bioreactors	Article	2018	10.1002/term.2531	co-culture MSC with ch
86	Moloudi et al	Inertial-Based Filtration Method for Removal of Microcarriers from Mesenchymal Stem Cell Suspensions	Article	2018	10.1038/s41598-018-31019-y	Cultivation carried out
87	Verbruggen et al	Bovine myoblast cell production in a microcarriers-based system	Article	2018	10.1007/s10616-017-0101-8	bovine myoblasts, no [illegible]
88	Costariol et al	Establishing the scalable manufacture of primary human T-cells in an automated stirred-tank bioreactor	Article	2019	10.1002/bit.27088	T cells, not MSCs (II)

89	Loubiere, C et al	Dimensional analysis and CFD simulations of microcarrier 'just-suspended' state in mesenchymal stromal cells bioreactors	Article	2019	10.1016/j.ces.2019.04.001	no Cultivierung only Si
90	Petry et al	Large-Scale Production of Size-Adjusted beta-Cell Spheroids in a Fully Controlled Stirred-Tank Reactor	Article	2022	10.3390/pr10050861	Diabetes, beta cells; n
91	Wyrobnik et al	Engineering characterization of the novel Bach impeller for bioprocessing applications requiring low power inputs	Article	2022	10.1016/j.ces.2021.117263	no cultivation (III)
92	Loffelholz et al	Dynamic Single-Use Bioreactors Used in Modern Liter- and m(3)-Scale Biotechnological Processes: Engineering Characteristics and Scaling Up	Article; Book Chapter	2014	10.1007/10_2013_187	Not Original Research
93	Rowley et al	DEVELOPMENT AND OPTIMIZATION OF XENO-FREE FED-BATCH STIRRED-TANK BIOREACTOR PROCESS FOR HMSC MANUFACTURING UTILIZING A DOE APPROACH	Meeting Abstract	2021		Not Original Research
94	Kirian et al	SCALE-UP OF A XENO-FREE FED-BATCH STIRRED-TANK BIOREACTOR PROCESS FOR HMSC MANUFACTURING	Meeting Abstract	2022		Not Original Research
95	Grein et al	Reprint of Multiphase mixing characteristics in a microcarrier-based stirred tank bioreactor suitable for human mesenchymal stem cell expansion	Reprint	2017	10.1016/j.procbio.2017.07.025	Not Original Research
96	King et al	Bioreactor development for stem cell expansion and controlled differentiation	Review	2007	10.1016/j.cbpa.2007.05.034	Not Original Research

97	Godara et al	Design of bioreactors for mesenchymal stem cell tissue engineering	Review	2008	10.1002/jctb.1918	Not Original Research
98	Naing et al	Three-dimensional culture and bioreactors for cellular therapies	Review	2011	10.3109/14653249.2011.556352	Not Original Research
99	Andrade et al	Stem cell bioengineering strategies to widen the therapeutic applications of haematopoietic stem/progenitor cells from umbilical cord blood	Review	2015	10.1002/term.1741	Not Original Research
100	Kumar et al	Large scale industrialized cell expansion: producing the critical raw material for biofabrication processes	Review	2015	10.1088/1758-5090/7/4/044103	Not Original Research
101	Schnitzler et al	Bioprocessing of human mesenchymal stem/stromal cells for therapeutic use: Current technologies and challenges	Review	2016	10.1016/j.bej.2015.08.014	Not Original Research
102	Mizukami et al	Technologies for large-scale umbilical cord-derived MSC expansion: Experimental performance and cost of goods analysis	Review	2018	10.1016/j.bej.2018.02.018	Not Original Research
103	Tsai et al	Influence of Microenvironment on Mesenchymal Stem Cell Therapeutic Potency: From Planar Culture to Microcarriers	Review	2020	10.3389/fbioe.2020.00640	Not Original Research
104	Couto et al	Expansion of human mesenchymal stem/stromal cells (hMSCs) in bioreactors using microcarriers: lessons learnt and what the future holds	Review	2020	10.1016/j.biotechadv.2020.107636	Not Original Research
105	Nath et al	Cell-Based Therapy Manufacturing in Stirred Suspension Bioreactor: Thoughts for cGMP Compliance	Review	2020	10.3389/fbioe.2020.599674	Not Original Research

106	Wyrobnik et al	Advances in human mesenchymal stromal cell-based therapies - Towards an integrated biological and engineering approach	Review	2020	10.1016/j.scr.2020.101888	Not Original Research
107	Petry et al	Impact of Bioreactor Geometry on Mesenchymal Stem Cell Production in Stirred-Tank Bioreactors	Review	2021	10.1002/cite.202100041	Not Original Research
108	Fuentes et al	Dynamic Culture of Mesenchymal Stromal/Stem Cell Spheroids and Secretion of Paracrine Factors	Review	2022	10.3389/fbioe.2022.916229	Not Original Research
109	Wu et al	Osteogenic Performance of Donor-Matched Human Adipose and Bone Marrow Mesenchymal Cells Under Dynamic Culture	Article	2015	10.1089/ten.tea.2014.0115	Cells were grown on g
110	Hanga et al	bioprocess for the expansion of	Article	2021	10.1002/bit.27842	bASCs, not MSCs (II)
111	Fujisawa et al	Development of cartilage tissue using a stirred bioreactor and human iPSC-derived limb bud mesenchymal cells	Article	2023	10.1016/j.bbrc.2023.149146	iPCSs differentiated to
112	Roberts et al	Computer controlled expansion of equine cord blood mesenchymal stromal cells on microcarriers in 3 L vertical-wheel® bioreactors	Article	2023	10.3389/fbioe.2023.1250077	Horse MSCs (II)
113	Roberts et al	Improved expansion of equine cord blood derived mesenchymal stromal cells by using microcarriers in stirred suspension bioreactors	Article	2019	10.1186/s13036-019-0153-8	Horse MSCs (II)
114	Schneider et al	Expansion of induced pluripotent stem cells under consideration of bioengineering aspects: part 1	Article	2025	10.1007/s00253-024-13372-3	iPSCs cultured, not MS
115	Teale et al	Expansion of induced pluripotent stem cells under consideration of bioengineering aspects: part 2	Article	2025	10.1007/s00253-024-13373-2	iPSCs cultured, not MS

116	Kamar et al	A Simple, Cost-Effective Microfluidic Device Using a 3D Cross-Flow T-Junction for Producing Decellularized Extracellular Matrix-Derived Microcarriers	Article	2025	10.1002/jbm.a.37873	hASC cultured, not MS
117	Painho et al	SCALABLE AND TRANSIENT GENE EXPRESSION IN MESENCHYMAL STROMAL CELLS EXPANDED IN STIRRED-TANK BIOREACTORS TOWARDS THE TREATMENT OF MYOCARDIAL INFARCTION	Meeting Abstract	2023		Not Original Research
118	Splan et al	Microcarrier-Based Xeno-Free Expansion of Human Mesenchymal Stromal Cells in a Single-Use Stirred-Tank Bioreactor	Meeting Abstract	2018		Not Original Research
119	Splan et al	MICROCARRIER-BASED XENO-FREE EXPANSION OF HUMAN MESENCHYMAL STROMAL CELLS IN A SINGLE-USE STIRRED-TANK BIOREACTOR	Meeting Abstract	2018		Not Original Research
120	Lenzini et al	DEVELOPING A MICROCARRIER STIRRED TANK PROCESS FOR LARGE-SCALE HMSC-EV PRODUCTION	Meeting Abstract	2022		Not Original Research
121	Maillot et al	Experimental study of transient particle suspension in bioreactors using a light attenuation technique	Article	2024	10.1016/j.ces.2023.119633	no cultivation (III)
122	Ichinohe et al	Proliferation, osteogenic differentiation, and distribution of rat bone marrow stromal cells in nonwoven fabrics by different culture methods	Article	2008	10.1089/ten.a.2007.0021	Rat Cells (II)

123	Boo et al	Expansion and preservation of multi	Article	2011	10.1007/s10856-011-4294-7	Rabit Cells (II)
124	Cherian et al	Biological Considerations in Scaling	Review	2020	10.3389/fphar.2020.00654	Not Original Research
125	Dufey et al	SCALABLE EXPANSION OF HUMA	Meeting Abstract	2017		Not Original Research
126	Ferrari et al	Investigation of Growth Conditions fo	Article	2014	10.1007/s12010-013-0586-3	Porcine Cells (II)
127	Herbst et al	Automated Production at Scale of In	Technical Note	2023	10.3390/pr11102938	Not Original Research
128	Li et al	Chemically crosslinked alginate poro	Article	2014	10.1002/jbm.b.33150	No MSCs, hepatocellu
129	Mawji et al	Challenges and opportunities in dow	Review	2022	10.1002/bit.28210	Not Original Research
130	Merten et al	Advances in cell culture: anchorage	Review	2015	10.1098/rstb.2014.0040	Not Original Research
131	Baksh et al	Adult human bone marrow-derived n	Article	2003	10.1016/S0301-472X(03)00106-1	No clear focus on MS
132	Cierpka et al	hMSC Production in Disposable Bior	Review	2013	10.1002/cite.201200151	Not Original Research
133	Loubière et al	Optimization of the Impeller Design f	Article	2019	10.1002/ceat.201900105	Computational Modelli
134	Roy et al	Stability of a biodegradable microcar	Article	2018	10.1039/c8tb01255e	Chemical Anlysis, no
135	Zeng et al	Effect of microcavitary alginate hydro	Article	2014	10.1016/j.msec.2013.09.003	Chondrocyte culture, n
136	Nienow et al	The Impact of Fluid Dynamic Stress	Review	2021	10.1002/cite.202000176	Not Original Research
137	Bonnaure et al	Bone Marrow Mesenchymal Stem C	Article	2016	10.1155/2016/7801781	Focuses on coc-ultivat
138	Ferrari et al	Limiting Cell Aggregation During Me	Article	2012	10.1002/btpr.1527	MSCs from pigs (II)
139	Narumi et al	Recovery of human mesenchymal st	Article	2020	10.1007/s10047-020-01186-9	MSCs grown statically,
140	Peter et al	Comparative study of in vitro expans	Article	2013	10.1007/s10529-012-1083-4	Rat Cells (II)
141	Warth et al	Fibrin Clots Maintain the Viability an	Article	2020	10.1097/CORR.0000000000001080	no agtitated cultivation
142	Chen et al	Cell culture design for homogeneous	Article	2024	10.1080/09205063.2023.2265623	mouse MSCs (II)

ion with B Lymphocytes, not MSC manufacturing (II)
